# The molecular mechanisms of peptidyl-prolyl *cis/trans* isomerase Pin1 and its relevance to kidney disease

**DOI:** 10.3389/fphar.2024.1373446

**Published:** 2024-04-22

**Authors:** Shukun Wu, Yurong Zou, Xiaoqiu Tan, Shuang Yang, Tangting Chen, Jiong Zhang, Xingli Xu, Fang Wang, Wei Li

**Affiliations:** ^1^ Department of Nephrology, Sichuan Provincial People’s Hospital, University of Electronic Science and Technology of China, Chengdu, China; ^2^ Key Laboratory of Medical Electrophysiology, Ministry of Education & Medical Electrophysiological Key Laboratory of Sichuan Province, Institute of Cardiovascular Research, Southwest Medical University, Luzhou, China; ^3^ Southwest Medical University, Luzhou, China; ^4^ State Key Laboratory for Innovation and Transformation of Luobing Theory, Key Laboratory of Cardiovascular Remodeling and Function Research, Chinese Ministry of Education, Chinese National Health Commission and Chinese Academy of Medical Sciences, Department of Cardiology, Qilu Hospital of Shandong University, Jinan, China; ^5^ Ultrasound in Cardiac Electrophysiology and Biomechanics Key Laboratory of Sichuan Province, Sichuan Provincial People’s Hospital, University of Electronic Science and Technology of China, Chengdu, China; ^6^ Department of Emergency Surgery, Sichuan Provincial People’s Hospital, University of Electronic Science and Technology of China, Chengdu, China

**Keywords:** Pin1, fibrosis, oxidative stress, autophagy, kidney disease

## Abstract

Pin1 is a member of the peptidyl-prolyl cis/trans isomerase subfamily and is widely expressed in various cell types and tissues. Alterations in Pin1 expression levels play pivotal roles in both physiological processes and multiple pathological conditions, especially in the onset and progression of kidney diseases. Herein, we present an overview of the role of Pin1 in the regulation of fibrosis, oxidative stress, and autophagy. It plays a significant role in various kidney diseases including Renal I/R injury, chronic kidney disease with secondary hyperparathyroidism, diabetic nephropathy, renal fibrosis, and renal cell carcinoma. The representative therapeutic agent Juglone has emerged as a potential treatment for inhibiting Pin1 activity and mitigating kidney disease. Understanding the role of Pin1 in kidney diseases is expected to provide new insights into innovative therapeutic interventions and strategies. Consequently, this review delves into the molecular mechanisms of Pin1 and its relevance in kidney disease, paving the way for novel therapeutic approaches.

## 1 Introduction

Kidney disease is an overarching term that encompasses a range of conditions resulting from diverse structural and functional damage to the kidneys, including acute kidney injury (AKI), chronic kidney disease (CKD), glomerular diseases, and diabetic nephropathy (DN) (Cavanaugh and Perazella, 2019; Luyckx et al., 2021). Due to challenges in early diagnosis and often overlooked symptoms, patients with kidney disease frequently progress to end-stage renal disease (ESRD), renal failure (RF), and a decline in renal function (Levey et al., 2003). The intricate pathogenesis of these conditions involves multiple molecular mechanisms and signaling pathways. Current treatments primarily rely on prolonged drug therapy, chronic dialysis, and kidney transplantation (Himmelfarb et al., 2020; Yan et al., 2021). However, these approaches often fail to yield satisfactory results. Therefore, it is important to elucidate the underlying mechanisms that contribute to the onset and progression of kidney disease to explore effective preventive and treatment interventions (O Toole and Sedor, 2014).

The functional properties of a protein are associated with its native-state topology, corresponding post-translational modifications, and signal-induced structural rearrangements (Matena et al., 2018). The cis/trans isomerization of peptide bonds involving any proteogenic amino acid, (Xaa)-Pro, is a rate-limiting step during protein structural rearrangements (Brandts et al., 1977). Peptidyl-proline isomerases (PPIases), which act as molecular switches, have previously been identified as participants in the acceleration of cis/trans isomerization. This process plays a crucial role in deactivating or activating enzymes and facilitating protein-protein interactions (Lang et al., 1987; Fischer et al., 1989; Andreotti, 2003). PPIases constitute a superfamily of molecular chaperones categorized by distinct topological structures and folds, including cyclophilins (CYPs), FK506-binding proteins (FKBPs), parvulins, and protein phosphatase (PPase) 2A phosphatase activators (PTPA) (Fischer et al., 1998; Jordens et al., 2006; Davis et al., 2008; Zhou and Lu, 2016). These enzymes can catalyze the isomerization of polypeptide bonds and play a role in regulating protein folding and function. This regulation controls the biological activities of numerous proteins and has been implicated in a wide range of diseases (Schmid, 1993; Fischer et al., 1998; Galat, 2003; Cao and Konsolaki, 2011; Nigro et al., 2013).

Pin1, also known as Protein NIMA 1, is an 18 kDa protein that belongs to the parvulin subfamily (Zhao et al., 2016). Numerous studies have demonstrated that Pin1-mediated isomerization plays a pivotal role in various biological processes, including the cell cycle, cell proliferation, apoptosis, signal transduction, transcription and splicing, immune response, and maintenance of the cytoskeleton (Esnault et al., 2007; Yeh and Means, 2007; Akiyama et al., 2009; Zhang J. H. et al., 2022). Furthermore, dysregulation of Pin1 is closely linked to the onset and progression of various diseases. These include neurodegenerative diseases, such as Alzheimer’s disease (AD), Parkinson’s disease (PD), amyotrophic lateral sclerosis (ALS), and Huntington’s disease (HD), as well as tumors, cardiometabolic diseases, and diseases related to viral infections (Sultana et al., 2006; Yeh and Means, 2007; Liang et al., 2018; Sun et al., 2020; Kato et al., 2022; Saeed and Piracha, 2023; Yang et al., 2023). Although the role of Pin1 in the aforementioned disorders have been extensively studied, its effect on kidney disease has received limited attention. Hence, we present a concise overview of Pin1, focusing on its structural biology and discussing the existing knowledge regarding its involvement in various kidney diseases.

## 2 Structure and biological function of Pin1

**FIGURE 1:**
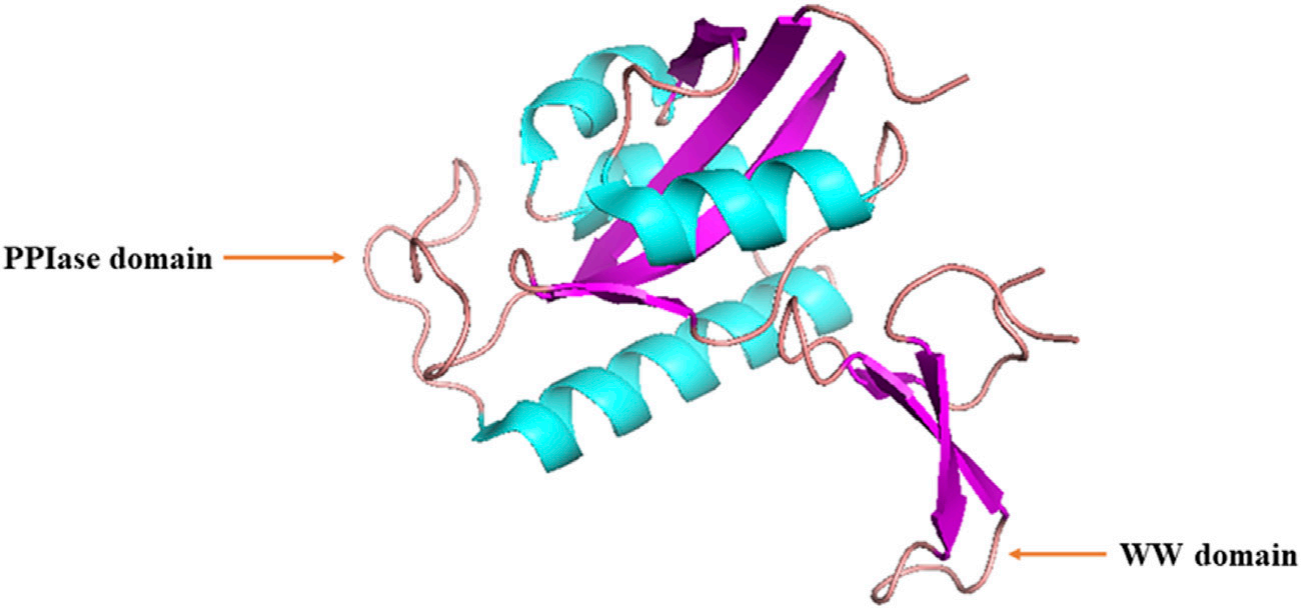
Structure of human Pin1 protein.

Lu et al. initially discovered that Ess1, the human ortholog of Pin1, serves as a regulatory protein involved in the growth, transcription, and mitosis of budding yeast (Hanes et al., 1989; Hani et al., 1995; Lu et al., 1996). Human *PIN1* is located on chromosome 19p13 and encodes a protein consisting of 163 amino acids (AAs) (Lu et al., 2009). Human Pin1 protein is a 163-AA peptidyl-coaminoyl cis/trans isomerase, consisting of an N-terminal WW (Trp-Trp) domain and a C-terminal PPIase domain with a central β-sheet (Figure 1) (Lu and Zhou, 2007; Nakatsu et al., 2016). These two domains are responsible for recognizing proteins containing phosphorylated Ser/Thr-Pro motifs and catalyzing cis-trans isomerization of the corresponding peptide bonds, respectively, forming a ‘double-check mechanism’ (Lu et al., 1996; Wintjens et al., 2001).

The upstream hydrophobic residues of the WW domain primarily include isoleucine, valine, phenylalanine, and/or tyrosine, whereas the downstream residues consist of arginine or lysine (Yaffe et al., 1997; Verdecia et al., 2000; Smet et al., 2005). The binding of the WW domain to pSer/Thr-Pro motifs is mainly maintained through hydrogen bonding and Van der Waals forces, with a binding dissociation constant at approximately 50 μm (Verdecia et al., 2000). The subcellular localization of Pin1 mainly depends on interactions between the WW domain and its corresponding substrates. *In vitro*, Pin1 primarily exists in the nucleus of cultured cells. However, in multiple cell types *in vivo*, Pin1 can be found in either the nucleus or cytoplasm, depending on the location of its substrates, which can be in either the nucleus or cytoplasm (Lu et al., 2002; Ayala et al., 2003; Liou et al., 2003).

The three-dimensional structure of a protein is determined by its AA sequence and folding patterns (Seckler and Jaenicke, 1992). During protein folding, the formation of disulfide bonds and the cis/trans isomerization of peptide bonds, especially those preceding proline residues, can influence protein modification (Warnke et al., 2014). Pro-directed protein kinases, including extracellular signal-regulated kinases (ERKs), c-Jun-N-terminal kinases (JNKs), p38 kinases, and Cyclin-dependent kinase 5 (CDK5), play significant roles in this process. While most peptide bonds are synthesized in the trans conformation, both cis and trans conformations are possible in peptidyl proline bonds because of the catalysis of peptide bond cis/trans isomerization by PPIases, including Pin1 (Matena et al., 2018).

Pin1 itself is subject to post-translational regulation (Lu et al., 2002; Lee et al., 2011; Rangasamy et al., 2012; Kim et al., 2015). The phosphorylation of Ser65 by polo-like kinase one increases the stability of Pin1 (Eckerdt et al., 2005). In contrast, small ubiquitin-like modifier (SUMO)-1 ubiquitinates Pin1, inhibiting its isomerase activity (Chen et al., 2013). Pin1 specifically catalyzes the isomerization of phosphorylated Ser/Thr-Pro motifs (Rostam et al., 2015). These cis/trans isomerization conformational changes result in stereoisomeric protein molecules with distinct structures and activities. These molecules then participate in the regulation of the activity, stability, phosphorylation level, and subcellular localization of target proteins, as well as interactions with other proteins (Yaffe et al., 1997). Consequently, Pin1 plays a pivotal role in regulating multiple biological processes. Pin1 also influences the activity of transcription factors such as β-catenin and NF-κB. It is involved in mitotic chromosome condensation through its interaction with topoisomerase (Topo) II alpha (Ryo et al., 2001; Ryo et al., 2002; Xu and Manley, 2007).

## 3 Representative pathophysiological roles of Pin1

To date, most studies have focused on investigating the pathophysiological roles of Pin1 and its inhibitors in cancer, neurodegenerative diseases, and cardiovascular diseases. These diseases have similar pathological features. In this section, we summarize the molecular mechanisms of action of Pin1 in different tissues and cell types beyond the kidney under various pathological conditions. These insights may provide novel approaches for the treatment of kidney diseases.

### 3.1 Representative inhibitors of Pin1

Pin1 inhibitors are chemical or natural compounds that hinder Pin1 activity and/or expression. Compounds such as Epigallocatechin-3-gallate, Juglone, cyclic peptide 11, KPT-6656, compound 8, and PiB have been shown to inhibit Pin1 enzymatic activity *in vitro* (Hennig et al., 1998; Urusova et al., 2011; Guo et al., 2014; Bedewy et al., 2017; Campaner et al., 2017; Lv et al., 2018). API-1 reduces Pin1 expression levels (Pu et al., 2018). All-trans retinoic acid and arsenic trioxide suppress both Pin1 activity and expression (Wei et al., 2015; Kozono et al., 2018). These Pin1 inhibitors are anticipated to offer therapeutic benefits to patients with a wide range of diseases.

### 3.2 Induction of fibrosis

**FIGURE 2:**
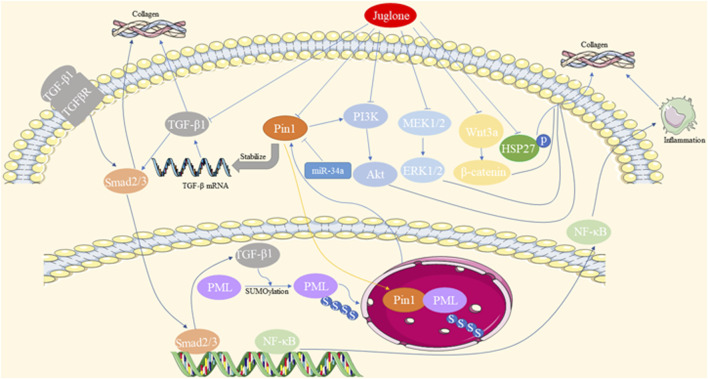
Mechanisms underlying the involvement of Pin1 in fibrosis.

Abnormal accumulation and deposition of extracellular matrix (ECM) proteins, as well as decreased expression and activation of matrix metalloproteinases (MMPs), contribute to fibrosis. Pin1 regulates the pathological process of fibrosis in multiple organs, including the heart, liver, and lungs. The mechanisms underlying the involvement of Pin1 in fibrosis are summarized in Figure 2.

Cardiac fibrosis is a common feature of end-stage heart diseases, such as diabetic cardiomyopathy (DCM), myocardial infarction (MI), and hypertrophic cardiomyopathy. Pin1, acting as a positive regulator of TGF-β1, colocalizes with promyelocytic leukemia protein (PML) and is involved in the TGF-β1/PML SUMOylation/Pin1 loop, all of which are upregulated in cardiac fibrosis (Liu et al., 2017; Wu et al., 2019). Pin1 inhibitors, such as Juglone, ginkgolic acid (inhibiting SUMO-1), miR-34a overexpression, and loureirin B, have been shown to alleviate cardiac fibrosis by reducing Pin1 expression and recruitment (Qiu et al., 2018; Zhang et al., 2021). Additionally, Juglone has been found to inhibit various signaling pathways, such as Pin1/TGF-β, TGF-β1/Smads, MEK1/2-ERK1/2, and α-SMA overproduction, in cardiac fibrosis induced by various heart diseases (Liu et al., 2016; Liu et al., 2017; Wu et al., 2018; Cheng et al., 2022). Juglone also disrupts the interaction between Pin1 and PML, destabilizing TGF-β mRNA and reducing p-Smad2/3 levels to protect against myocardial fibrosis (Wu et al., 2019).

Liver fibrosis is characterized by the excessive accumulation of ECM proteins, which can lead to hepatic cirrhosis and hepatoma development. Pin1 plays an essential role in fibrotic accumulation in liver pathologies such as non-alcoholic steatohepatitis (NASH). In clinical research, Pin1 has emerged as an independent predictor of liver fibrosis, with serum Pin1 levels being correlated with the histopathological stages of liver fibrosis in NASH (Cengiz et al., 2014). Additionally, genetic polymorphisms in PIN1 have been linked to the risk of hepatitis B virus-related liver cirrhosis among HBV-infected patients in Guangxi, China (Huang et al., 2018). Pin1 has been found to be upregulated in NASH mouse models and promotes liver fibrosis through various pathways (Nakatsu et al., 2012; Yang et al., 2014; Kim et al., 2016). Pin1 facilitates the association of Smad3 with WW domain-containing transcription regulators (TAZ). Pin1 downregulation, either through its inhibitors or siRNA, reduces the expression of collagen 1a1/2, α-SMA, and fibronectin, indicating its pivotal role in ECM component production in hepatic stellate cells (HSCs) and liver fibrosis (Aoyama et al., 2023). Moreover, Juglone suppresses liver fibrosis via the downregulation of the Wnt3a/β-catenin pathway in carbon tetrachloride-induced liver injury (Kim et al., 2016). Pin1 knockout (KO) mice are resistant to methionine choline-deficient (MCD) diet-induced NASH and fibrosis accumulation partially via the downregulation of peroxisome proliferator-activated receptor alpha (PPARα), as well as inhibition of NF-κB activation and its downstream inflammatory cytokines (Nozaki et al., 2009; Nakatsu et al., 2012). In dimethylnitrosamine (DMN)-induced mice livers, Juglone ameliorates liver fibrosis by reducing the expression of TGF-β1, α-SMA, and plasminogen activator inhibitor-I (PAI-1) through the downregulation of Erk-PI3K/Akt and p-Smad2/3 activation (Yang et al., 2014).

Pulmonary fibrosis is a chronic, progressive lung disease that can be triggered by various etiological factors, ultimately leading to the disruption of lung architecture. Studies have demonstrated that Pin1 plays a regulatory role in lung fibrosis, both in cases of acute lung injury and chronic asthma (Shen et al., 2008; Shen et al., 2012). According to Shen et al., Pin1 deficiency suppresses the expression of collagen I/III/V, PAI-1, and TGF-β1. This reduction in expression results in decreased interstitial pulmonary fibrosis owing to the accumulation of excessive cytoplasmic Smad6. This, in turn, leads to the reduction of TGF-β-induced p-Smad3 activation and its nucleus translocation, contributing to the mitigation of pulmonary fibrosis in acute bleomycin lung injury (Shen et al., 2012). Furthermore, the inhibition of Pin1 by Juglone has been shown to further reduce the expression of collagen I/III and stabilize TGF-β1 mRNA in pulmonary eosinophils. This action prevents airway fibrosis in a chronic asthma mouse model (Shen et al., 2008).

In conclusion, Pin1 regulates fibrosis in the heart, liver, and lungs by influencing ECM accumulation and molecular pathways. These findings highlight Pin1 as a potential target for fibrotic disease intervention.

### 3.3 Effects of oxidative stress

**FIGURE 3:**
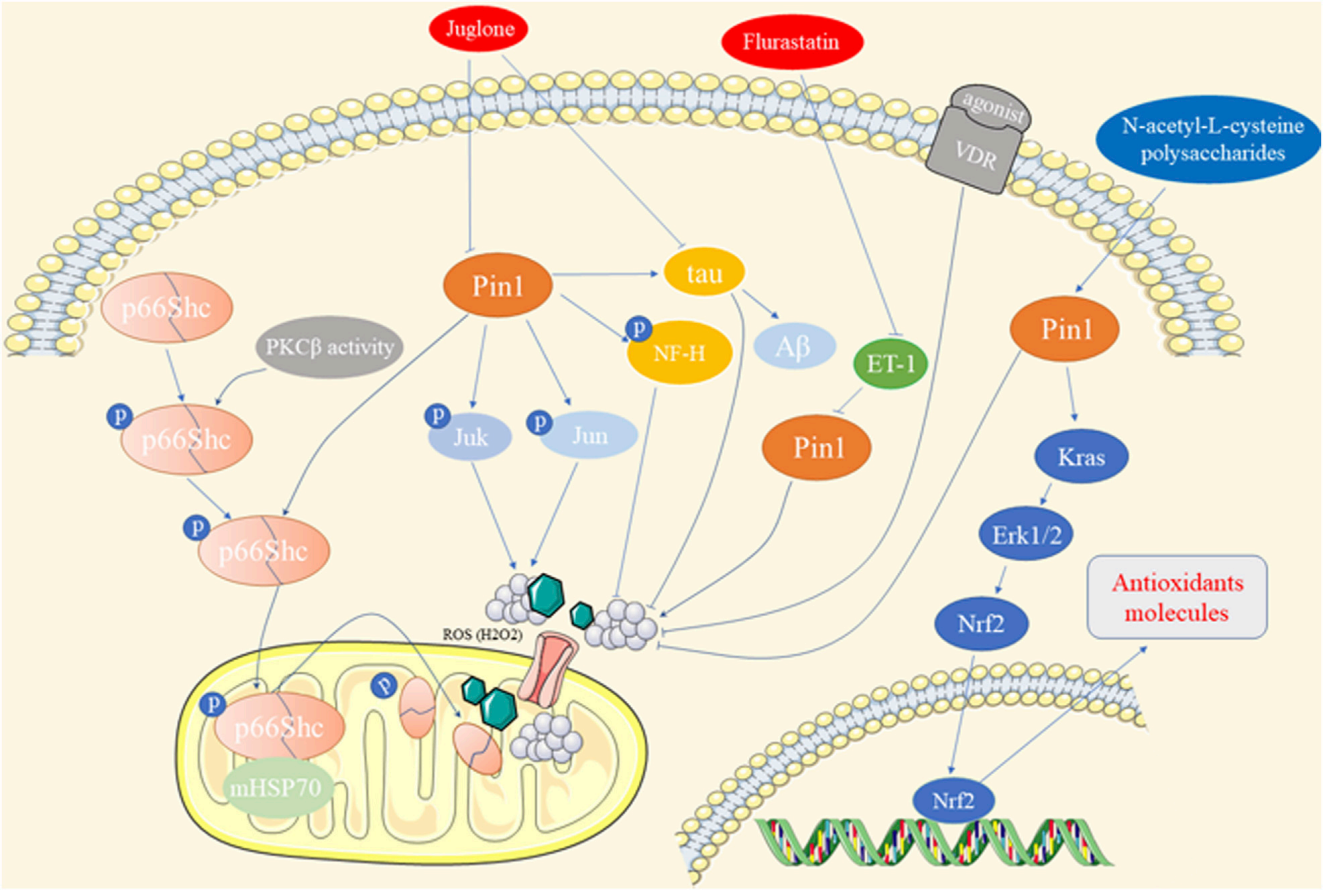
The role of Pin1 in oxidative stress-related pathology.

Oxidative stress arises from an imbalance between free radical production and antioxidant defenses, contributing to various diseases, such as diabetes, neurodegeneration, cardiovascular issues, and cancer (Sun et al., 2023). Pin1 has been shown to play a role in oxidative stress-related pathologies, as depicted in Figure 3.

Francesco et al. demonstrated that Pin1 exacerbates hyperglycemia-induced reactive oxygen species (ROS) production and mitochondrial oxidative stress in human aortic endothelial cells (HAECs) through the translocation of p66Shc, a prooxidant adaptor (Pinton et al., 2007; Paneni et al., 2015). Suppression of Pin1, through gene silencing, Juglone treatment, Vitamin D receptor (VDR) agonists, or bromocriptine-QR, alleviates chronic oxidative stress and vascular dysfunction induced by diabetes (Paneni et al., 2015; Costantino et al., 2016; Zhang et al., 2018; Cincotta et al., 2022). Similar effects of Pin1 on p66Shc translocation and ROS accumulation were observed in intestinal ischemia/reperfusion (I/R) injury and hippocampal neuronal oxidative stress (Zhu et al., 2014; Feng et al., 2017). Furthermore, Pin1 promotes mitochondrial oxidative stress in conditions such as ISO-induced cardiac fibrosis, alcoholic cardiomyopathy, and cardiomyocyte hypertrophy (Sakai et al., 2014; Wang et al., 2016; Wu et al., 2018). Fluvastatin mitigates endothelin-1-induced cardiomyocyte hypertrophy by suppressing Pin1 activity through the regulation of p-JNK and c-Jun (Park et al., 2012; Sakai et al., 2014). In contrast, Pin1 upregulates antioxidant response element-driven genes via the Kras/ERK/NRF2 axis in pancreatic cancer cells (Oke et al., 2020). Pin1 overexpression reduces ROS production and hepatic oxidative stress in arsenic- and CCl_4_-induced hepatotoxicity (Zhang H. et al., 2022). N-acetyl-L-cysteine and polysaccharides from *Enteromorpha prolifera* restore Pin1 levels, reducing oxidative stress and protecting against hepatic injury (Guo et al., 2020; Zhang H. et al., 2022).

Oxidative stress induced by amyloid-β peptide (Aβ) plays a crucial role in AD in humans and animal models. Pin1 was found to colocalize with Tau, regulating its phosphorylation/dephosphorylation and inducing Aβ production (Robinson et al., 2011). Redox proteomic analyses revealed elevated levels of Pin1 in a mouse model of AD (Robinson et al., 2011). Juglone prevents Tau dephosphorylation at Thr-231, reducing oxidative stress in AD (Galas et al., 2006). Green tea catechins improve the AD phenotype by increasing Pin1 activity (Lim et al., 2013). Additionally, Pin1 enhances the phosphorylation of high-molecular-weight neurofilament protein (NF-H) through proline-directed kinases such as JNK3 in neurodegenerative disorders, including AD and amyotrophic lateral sclerosis (Rudrabhatla et al., 2008). However, some studies have reported lower Pin1 expression and activity, along with oxidative modifications, is associated with mild cognitive impairment, the hippocampus in AD, and adriamycin-induced cognitive dysfunction (Butterfield et al., 2006; Sultana et al., 2006; Joshi et al., 2010).

Given the varying effects of Pin1 on oxidative stress-related processes, its role in kidney disease is a compelling and significant area of research.

### 3.4 Effects of autophagy

Autophagy is a crucial process that maintains cellular homeostasis by breaking down damaged organelles, abnormal proteins, and pathogens within autophagosomes. These autophagosomes fuse with lysosomes for bulk degradation (Chao et al., 2022).

In the context of cadmium exposure, the expression of Pin1 shows interesting effects on autophagy. Previous investigation has shown that exposure of oral squamous cell carcinoma to cadmium (IC50 = 45 μM) decreases the expression of Pin1. Inhibiting Pin1 with siRNA results in suppressed autophagy, which is inversely associated with p-Akt and p-Ser-GSK3αβ levels (So et al., 2015). Similarly, Pin1 levels decrease following cadmium exposure in human hepatoma cells (HepG2) with an IC50 ≥ 6 μΜ, and the inhibition of Pin1 induces autophagy, likely through the upregulation of p-Ser-GSK3αβ (So and Oh, 2015). This Pin1-autophagy relationship has also been observed in senescent auditory hair cells, where Pin1 inhibition leads to LC3 upregulation and p62 downregulation (Lv et al., 2022). While changes in GSK3αβ expression levels have no impact on cadmium-induced Pin1 levels in HepG2, the inhibition of GSK-3β in mouse livers leads to Pin1 activation, suggesting that GSK-3β might mediate the role of Pin1 in autophagy (Zhang J. H. et al., 2022; Chao et al., 2022). Additionally, in tamoxifen-resistant breast cancer, elevated levels of Pin1 enhance its interaction with p-MEK1/2, resulting in increased E2F-4- and Egr-1-driven LC-3 expression (Namgoong et al., 2010). This suggests a positive correlation between Pin1 levels and LC-3 expression, a representative indicator of autophagy, in estrogen receptor alpha-positive breast cancer.

**FIGURE 4:**
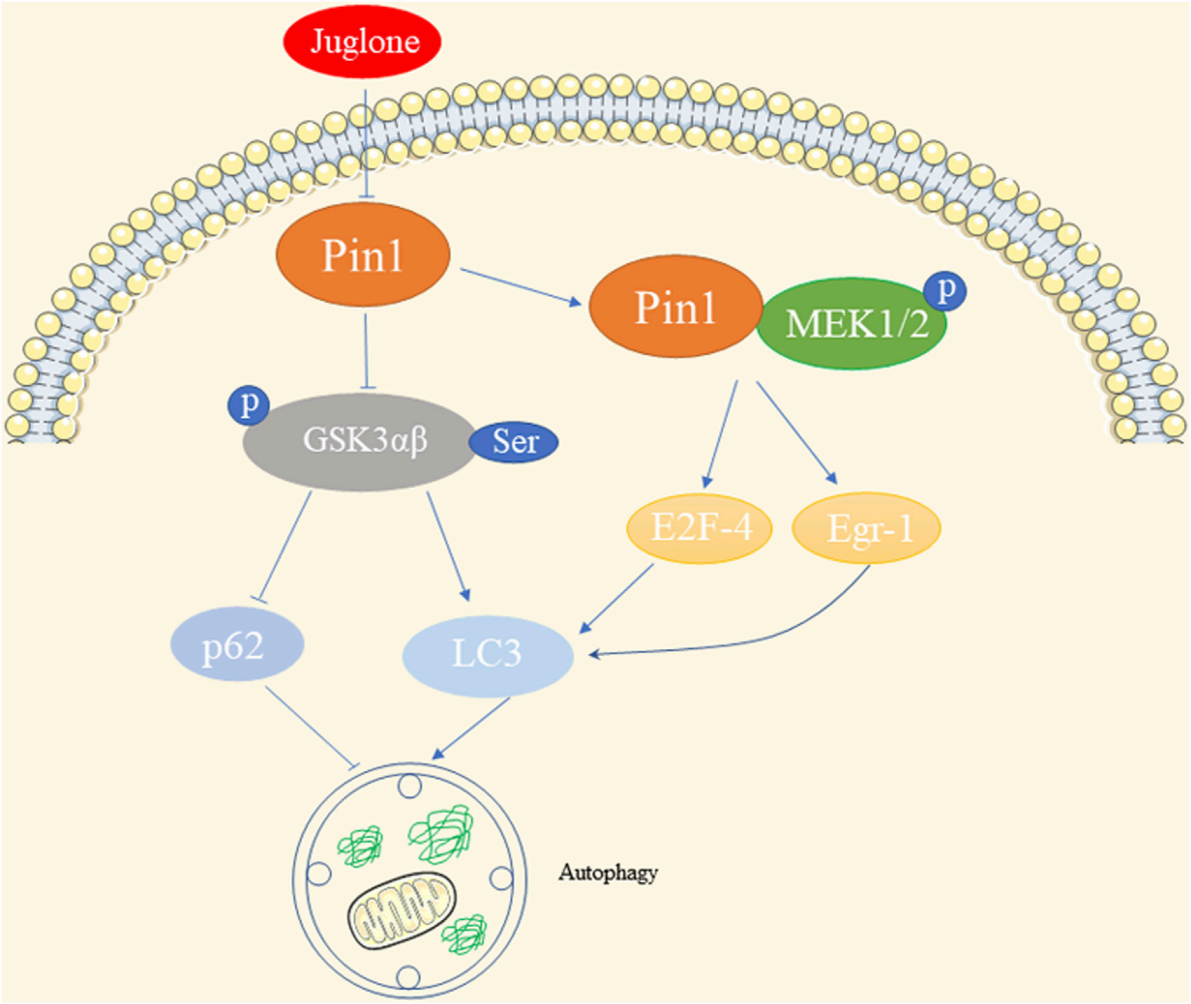
Effects of Pin1 on autophagy.

In summary, cadmium exposure impacts Pin1 and subsequently influences autophagy through pathways involving p-Akt, GSK3αβ, and LC-3 in different cell types. The diverse effects of Pin1 on autophagy, as illustrated in Figure 4, justify the need for further exploration in various kidney diseases.

## 4 Effects of Pin1 on kidney disease

**FIGURE 5:**
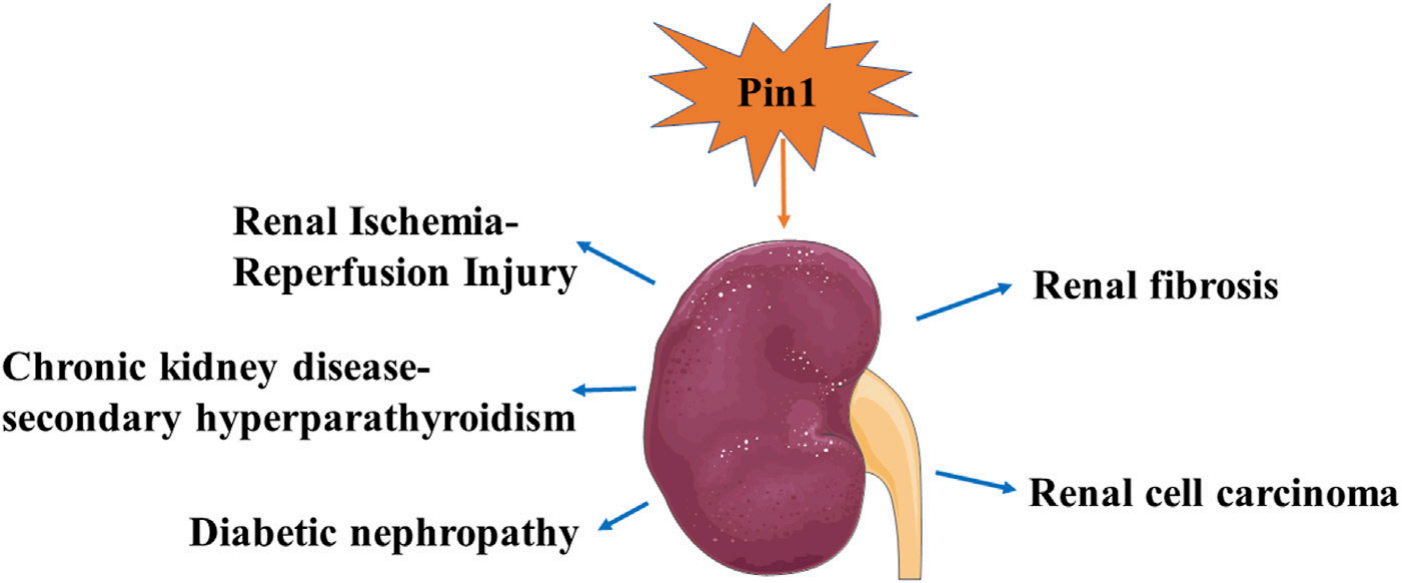
Recent research findings and advancements for Pin1 in kidney diseases.

The role of Pin1 in mouse kidney development and kidney disease is of significant interest. Li et al. have highlighted the critical effects of Pin1 on embryonic mouse kidney development. They found that Pin1 is expressed in ureteric bud derivatives and mediates the function of protein kinase-X (PRKX), a regulator of epithelial morphogenesis, through the WW domain of Pin1 (Li et al., 2009). In contrast, Pin1 has been linked to the occurrence and progression of various kidney diseases (Thorpe et al., 2001; Hassan et al., 2022a; Patel et al., 2022). Genetic or pharmacological inhibition of Pin1 may help reduce renal injury by mitigating kidney fibrosis, oxidative stress, and apoptosis (Shen et al., 2016; Zhao et al., 2021). We have summarized the latest research findings and advancements regarding Pin1 in kidney disease in Figure 5 and Table 1.

**TABLE 1:** Recent research findings and advancements for Pin1 in kidney diseases.

Kidney disease	Humans/Animals/Cells	Treatments	Pin1 role in diagnostics or prognostics
Acute kidney injury (AKI)	I/R SD rats	Juglone or siPin1	-alleviate renal injuries in structure and function
	HK-2 cells		-increase SOD expression level
			-reduce expression levels of MDA, ROS and H_2_O_2_
			-reduce the expression levels of p38 MAPK
	I/R SD rats	Juglone or siPin1	-protect renal I/R injury from cellular damage
	HK-2 cells		-reduce expression levels of GRP78, elF2ɑ and CHOP
			-down-regulate Nrf-2/HO-1 pathways
Chronic kidney disease-secondary hyperparathyroidism (CKD-SHPT)	CKD SD rats	Pin1 inhibition	-decrease KSRP-PTH mRNA interactions
	transfected cells		-increase serum PTH
	Pin1^−/−^ mice		- increase PTH mRNA levels
	patients with ESRD	No treatment	-lower Pin1 expression levels
			-associate with nodular hyperplasia
			-influence PTH secretion
	Chinese Han patients with ESRD	No treatment	-PIN1 promoter polymorphism as vital genetic determinants in etiopathogenesis
			-Pin1 as biomarkers of susceptibility to CKD-SHPT
	Indian patients with ESRD	No treatment	-PIN1 promoter polymorphism as vital genetic determinants in etiopathogenesis
			-Pin1 as biomarkers of susceptibility to CKD-SHPT
Diabetic nephropathy (DN)	NA/STZ/Suc mice	Canagliflozin (SGLT2 inhibitors)	-normalize the upregulated Pin1 expression levels
			-decrease the fibrosis and inflammatory cytokines
	HK-2 cells	HG and siPin1	-reduce HG-induced p66Shc activation
			-reduce HG-induced p66Shc translocation into the mitochondrial compartment
Renal fibrosis	Mice with T2DM	HG	-Pin1 expression increased along with fibrosis of kidney
	mice with high phosphate	Pin1^−/−^	-inhibit the ECM deposition and renal tubulointerstitial fibrosis through non-Smad pathways
	Lewis rats with UUO	Juglone	-decrease deposition of matrix, renal fibrosis, EMT, oxidative stress through p-Smad2 and p-HSP27 pathways
			-attenuate kidney fibrogenesis via Pin1-independent mechanisms
Renal cell carcinoma (RCC)	RCC	No treatment	-binding of Pin1 to TIS21 is reduced in the RCC
			-result in cell death
	mouse embryo fibroblasts	Pin1 deficiency	-lead to genomic instability
			-extensive tumorigenesis induced by the Ras oncogene
	human clear cell RCC tumors	Pin1 overexpression	-PIN1 gene is frequently deleted on chromosome 19p13.2 and under-expressed in human clear cell RCC tumors
	a xenograft tumor model		-Pin1 attenuates tumor growth through proliferation inhibition and apoptosis induction dependent on functional p53 in a xenograft tumor model
	nephroblastoma in a young C57BL/6 mouse	deletion of the Pin1 gene	-deletion of the Pin1 gene couples with Trp53 abnormality
	mice bearing 786-O cell-derived tumor	Pin1 inhibitor with PiB or Juglone	-suppress the PML protein ubiquitination and degradation
			-inactivated mTOR-HIF signaling pathway to enhance ccRCC suppression

### 4.1 Renal I/R injury

AKI is a heterogeneous condition characterized by a rapid decline in renal function over a short period (several hours to several days) and is typically accompanied by a sudden increase in serum creatinine and/or reduced urine output (Bellomo et al., 2012; Levey and James, 2017). Clinically, AKI is associated with high mortality and disability rates. The pathological processes underlying AKI can be summarized as follows: first, inadequate renal blood flow leads to cellular damage, apoptosis, and necrosis; second, inflammatory cell infiltration and a sustained inflammatory response play a crucial role in I/R injury and exacerbate kidney injury (Peng et al., 2023); and third, the accumulation of renal toxic substances, such as hemoglobin and myoglobin, leads to an overload of protein reabsorption in the kidneys, excessive production of reactive oxygen species (ROS), and even severe oxidative stress and endoplasmic reticulum (ER) stress. Currently, research on Pin1 in AKI is limited to I/R kidney injury, and further studies are needed to explore its role in other types of AKI.

Renal I/R injury is a significant risk factor for AKI. Zhao et al. demonstrated that Pin1 inhibition, achieved either through Pin1 knockdown or Juglone treatment, alleviates structural and functional renal injury, increases SOD expression, and reduces the levels of MDA, ROS, and H_2_O_2_, which are indicators of oxidative stress. These effects are linked to decreased p38 MAPK expression in male Sprague-Dawley (SD) rats with I/R-induced AKI (Zhao et al., 2021). Furthermore, Yu et al. reported that Juglone or si-Pin1 protects against renal I/R-induced AKI by mitigating cellular damage and reducing the expression of ER stress markers, including GRP78, eIF2α, and CHOP. This protective effect is achieved by the downregulation of the Nrf-2/HO-1 pathway (Yu et al., 2022). Future studies should investigate the association between Pin1 and additional pathological processes triggered by I/R-related AKI as well as AKI resulting from sepsis or other nephrotoxic events.

### 4.2 Chronic kidney disease-secondary hyperparathyroidism (CKD-SHPT)

**FIGURE 6:**
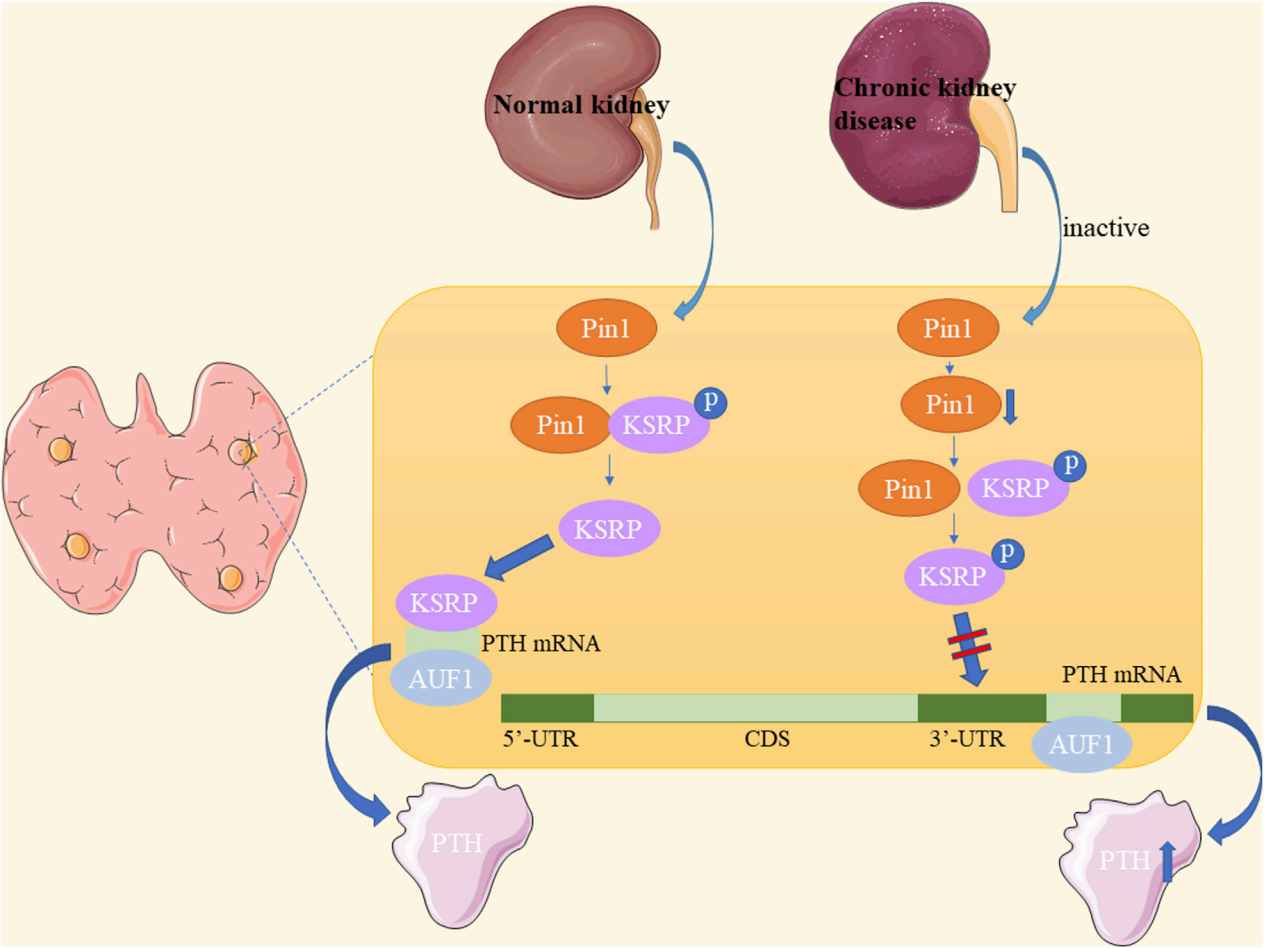
Underlying mechanisms of Pin1 in chronic kidney disease-secondary hyperparathyroidism (CKD-SHPT).

CKD is characterized by chronic structural and functional impairment of the kidneys lasting at least 3 months, resulting from various causes such as primary and secondary glomerulonephritis, tubular injury, and vascular lesions of the kidney (Lederer and Ouseph, 2007; Fraser, 2009). Pin1 is a crucial regulator of parathyroid hormone (PTH) stability (Kumar, 2009). In CKD, Pin1 isomerization becomes inactive, leading to reduced Pin1-mediated dephosphorylation and conformational changes of K-homology splicing regulatory protein (KSRP). Consequently, KSRP fails to effectively bind to PTH mRNA (Kilav-Levin et al., 2020; Hassan et al., 2022a; Hassan et al., 2022b). This results in decreased PTH mRNA destabilization by KSRP, whereas stabilization by adenosine-uridine-rich binding factor 1 (AUF1) increases, leading to elevated PTH mRNA levels and stability (Kilav-Levin et al., 2020) (Figure 6).

Furthermore, Morris et al. demonstrated in CKD rats and Pin1^−/−^ mice that Pin1 inhibition reduces KSRP-PTH mRNA interactions, leading to increased serum PTH and PTH mRNA levels in CKD-SHPT (Nechama et al., 2009). Irena et al. also found that lower Pin1 expression levels are associated with nodular hyperplasia and influence PTH secretion in patients with end-stage renal disease (ESRD) (Tycova et al., 2016). Clinically, the PIN1 promoter polymorphism may be a significant genetic determinant in the etiopathogenesis of CKD-SHPT and may serve as a biomarker of susceptibility in the Chinese Han population and Indian patients with CKD (Zhao et al., 2017; Patel et al., 2022). Therefore, Pin1 plays a role in the pathogenesis of SHPT in patients with CKD. Pin1 inactivation in CKD affects PTH mRNA stability, and future research may uncover a broader role of Pin1 in CKD pathogenesis.

### 4.3 Diabetic nephropathy (DN)

DN is the most common cause of RF in patients with diabetes mellitus (DM) and often necessitates dialysis (Zhu et al., 2022). Worldwide in 2019, there were 2.62 million new cases, 134.58 million patients, 13.09 million disability-adjusted life years (DALYs), and 405.99 thousand deaths attributed to CKD-DM (Deng et al., 2021). Moreover, the incidence of DN in patients with DM is as high as 20%–50% (Dua et al., 2021). Type 2 diabetes is the most common type of diabetes and its pathological processes involve fibrotic deposition, oxidative stress, inflammation, apoptosis, and autophagy (Zhu et al., 2022).

Masa-Ki et al. demonstrated that Pin1 is involved in the protective effects of sodium-glucose co-transporter 2 (SGLT2) inhibitors in the kidneys of mice with hyperglycemia induced by nicotinamide, streptozotocin, and a high-sucrose diet (NA/STZ/Suc). Canagliflozin normalizes elevated Pin1 expression levels, subsequently leading to a reduction in fibrosis and inflammatory cytokines (Inoue et al., 2019). Furthermore, high glucose (HG) increases the interaction between Pin1 and phosphorylated p66Shc in the mitochondria, resulting in increased oxidative stress and apoptosis in HK-2 cells. Reduction in Pin1 expression leads to decreased p66Shc activation and its translocation into the mitochondria in response to HG (Sun et al., 2010). These effects are also linked to increased HG-induced angiotensin II (Ang II) synthesis (Efrati et al., 2009; Sun et al., 2010).

### 4.4 Renal fibrosis

The maintenance of ECM homeostasis is crucial for renal function. Disruption of this balance, particularly in the epithelial-to-mesenchymal transition (EMT), is implicated in initiating and advancing renal fibrosis, especially in CKD (Liu, 2011; He et al., 2013). As mentioned earlier, Pin1 expression increases with kidney fibrosis in type 2 DM mice (Inoue et al., 2019). High phosphate levels, another risk factor for kidney fibrosis, exacerbate the decline in kidney function. Shen et al. discovered that Pin1 deficiency inhibits ECM deposition and renal tubulointerstitial fibrosis through non-Smad pathways in mice subjected to a high phosphate diet (HPD) (Shen et al., 2016). Juglone reduces matrix deposition, EMT, oxidative stress and attenuated renal fibrosis via the p-Smad2 and p-heat shock protein 27 (HSP27) pathways in Lewis rats with unilateral ureteral occlusion (UUO) (Reese et al., 2010). Pin1 regulates the pathological process of DN and exerts an antifibrotic effect during the deterioration of renal function under conditions related to high phosphate- and occlusion-induced kidney injury. Therefore, Pin1 plays a crucial role in maintaining ECM balance and preventing renal fibrosis.

### 4.5 Renal cell carcinoma (RCC)

RCC, also referred to as renal adenocarcinoma, is a malignancy arising from the urinary tubular epithelial system within the renal parenchyma (Linehan and Ricketts, 2019). RCC encompasses multiple subtypes originating from various segments of the urinary tubules, excluding tumors arising in the renal interstitium or pelvis (Wettersten et al., 2017).

Pin1 deficiency can lead to genomic instability and increased tumorigenesis. PIN1 is frequently deleted and under-expressed in human clear cell RCC tumors. The interaction between Pin1 and TIS21, a tumor suppressor that is highly expressed in normal proximal tubules of the kidney but is lost in RCC, leads to cell death. This suggests potential implications for Pin1 in RCC development (Struckmann et al., 2004; Lim et al., 2008). Previous studies have demonstrated that Pin1 deficiency can result in genomic instability and extensive tumorigenesis when induced by the Ras oncogene in mouse embryo fibroblasts (Yeh et al., 2006). Brian et al. reported that the PIN1 gene is often deleted on chromosome 19p13.2 and is under-expressed in human clear cell RCC tumors. They found that Pin1 attenuates tumor growth by inhibiting proliferation and inducing apoptosis in a xenograft tumor model, which depends on functional p53 (Teng et al., 2011). Vittoria et al. reported the development of nephroblastoma in a young C57BL/6 mouse with a Pin1 deletion coupled with a Trp53 abnormality. This discovery provides novel insights into the relationship between Pin1 and nephroblastoma tumor pathogenesis (Castiglioni et al., 2013).

In contrast, the Pin1 inhibitors PiB and Juglone suppress the ubiquitination and degradation of promyelocytic leukemia (PML) proteins. Additionally, they inactivate the mTOR-HIF signaling pathway, leading to enhanced suppression of clear cell RCC in mice bearing tumors derived from 786-O cells (Lin et al., 2014). Therefore, Pin1 appears to play a significant role in the development of RCC.

## 5 Conclusion

Recently, multiple studies have highlighted the significant roles of Pin1 and its inhibitors in the pathophysiology of kidney diseases. Pin1 has been shown to contribute to pathological processes such as fibrosis, oxidative stress, and autophagy in various tissues and cell types, including the kidney. We present existing evidence for the involvement of Pin1 in conditions such as AKI, CKD-SHPT, DN, renal fibrosis, and RCC. However, the regulatory role of Pin1 in other kidney diseases remains unclear. These diseases include kidney transplantation and other forms of AKI such as sepsis, cisplatin nephrotoxicity, lupus nephritis, and IgA nephropathy. Furthermore, it is crucial to emphasize the need for both clinical and preclinical trials to elucidate the effects of Pin1 on kidney disease. In conclusion, Pin1 plays a vital role in the pathogenesis of kidney diseases, and modulating its expression (either upregulation or downregulation) may offer effective therapeutic strategies for various kidney disorders. In the future, regulating the expression of Pin1 is expected to be beneficial in the treatment of kidney diseases. For example, gene therapy may be used to modify Pin1 expression for treating kidney disease or inhibitors of Pin1 may be used to treat AKI and CKD. This opens new avenues for the early prevention and treatment of kidney disease.

## References

[B1] Akiyama H. Gotoh A. Shin R. W. Koga T. Ohashi T. Sakamoto W. (2009). A novel role for hGas7b in microtubular maintenance: possible implication in tau-associated pathology in Alzheimer disease. J. Biol. Chem. 284, 32695–32699. 10.1074/jbc.M109.035998 19801671 PMC2781685

[B2] Andreotti A. H. (2003). Native state proline isomerization: an intrinsic molecular switch. Biochemistry 42, 9515–9524. 10.1021/bi0350710 12911293

[B3] Aoyama S. Kido Y. Kanamoto M. Naito M. Nakanishi M. Kanna M. (2023). Prolyl isomerase Pin1 promotes extracellular matrix production in hepatic stellate cells through regulating formation of the Smad3-TAZ complex. Exp. Cell Res. 425, 113544. 10.1016/j.yexcr.2023.113544 36906101

[B4] Ayala G. Wang D. Wulf G. Frolov A. Li R. Sowadski J. (2003). The prolyl isomerase Pin1 is a novel prognostic marker in human prostate cancer. Cancer Res. 63, 6244–6251. 14559810

[B5] Bedewy W. Liao H. Abou-Taleb N. A. Hammad S. F. Nasr T. Pei D. (2017). Generation of a cell-permeable cycloheptapeptidyl inhibitor against the peptidyl-prolyl isomerase Pin1. Org. Biomol. Chem. 15, 4540–4543. 10.1039/c7ob00430c 28517007 PMC5520971

[B6] Bellomo R. Kellum J. A. Ronco C. (2012). Acute kidney injury. Lancet 380, 756–766. 10.1016/S0140-6736(11)61454-2 22617274

[B7] Brandts J. F. Brennan M. Lung-Nan L. (1977). Unfolding and refolding occur much faster for a proline-free proteins than for most proline-containing proteins. Proc. Natl. Acad. Sci. U. S. A. 74, 4178–4181. 10.1073/pnas.74.10.4178 22075 PMC431901

[B8] Butterfield D. A. Poon H. F. St Clair D. Keller J. N. Pierce W. M. Klein J. B. (2006). Redox proteomics identification of oxidatively modified hippocampal proteins in mild cognitive impairment: insights into the development of Alzheimer's disease. Neurobiol. Dis. 22, 223–232. 10.1016/j.nbd.2005.11.002 16466929

[B9] Campaner E. Rustighi A. Zannini A. Cristiani A. Piazza S. Ciani Y. (2017). A covalent PIN1 inhibitor selectively targets cancer cells by a dual mechanism of action. Nat. Commun. 8, 15772. 10.1038/ncomms15772 28598431 PMC5472749

[B10] Cao W. Konsolaki M. (2011). FKBP immunophilins and Alzheimer's disease: a chaperoned affair. J. Biosci. 36, 493–498. 10.1007/s12038-011-9080-7 21799260

[B11] Castiglioni V. De Maglie M. Queliti R. Rustighi A. Del Sal G. Radaelli E. (2013). Immunohistochemical characterization of a renal nephroblastoma in a trp53-mutant and prolyl isomerase 1-deficient mouse. J. Toxicol. Pathol. 26, 423–427. 10.1293/tox.2013-0021 24526816 PMC3921926

[B12] Cavanaugh C. Perazella M. A. (2019). Urine sediment examination in the diagnosis and management of kidney disease: core curriculum 2019. Am. J. Kidney Dis. 73, 258–272. 10.1053/j.ajkd.2018.07.012 30249419

[B13] Cengiz M. Ozenirler S. Yucel A. A. Yilmaz G. (2014). Can serum pin1 level be regarded as an indicative marker of nonalcoholic steatohepatitis and fibrotic stages? Digestion 90, 35–41. 10.1159/000365415 25170559

[B14] Chao X. Wang S. Fulte S. Ma X. Ahamed F. Cui W. (2022). Hepatocytic p62 suppresses ductular reaction and tumorigenesis in mouse livers with mTORC1 activation and defective autophagy. J. Hepatol. 76, 639–651. 10.1016/j.jhep.2021.10.014 34710483 PMC8858859

[B15] Chen C. H. Chang C. C. Lee T. H. Luo M. Huang P. Liao P. H. (2013). SENP1 deSUMOylates and regulates Pin1 protein activity and cellular function. Cancer Res. 73, 3951–3962. 10.1158/0008-5472.CAN-12-4360 23633483 PMC3818121

[B16] Cheng M. Yang Z. Li R. Wu G. Zhang C. (2022). Loureirin B alleviates cardiac fibrosis by suppressing Pin1/TGF-β1 signaling. Eur. J. Pharmacol. 918, 174791. 10.1016/j.ejphar.2022.174791 35093323

[B17] Cincotta A. H. Cersosimo E. Alatrach M. Ezrokhi M. Agyin C. Adams J. (2022). Bromocriptine-QR therapy reduces sympathetic tone and ameliorates a pro-oxidative/pro-inflammatory phenotype in peripheral blood mononuclear cells and plasma of type 2 diabetes subjects. Int. J. Mol. Sci. 23, 8851. 10.3390/ijms23168851 36012132 PMC9407769

[B18] Costantino S. Paneni F. Luscher T. F. Cosentino F. (2016). Pin1 inhibitor Juglone prevents diabetic vascular dysfunction. Int. J. Cardiol. 203, 702–707. 10.1016/j.ijcard.2015.10.221 26583846

[B19] Davis T. L. Walker J. R. Ouyang H. MacKenzie F. Butler-Cole C. Newman E. M. (2008). The crystal structure of human WD40 repeat-containing peptidylprolyl isomerase (PPWD1). FEBS J. 275, 2283–2295. 10.1111/j.1742-4658.2008.06381.x 18397323

[B20] Deng Y. Li N. Wu Y. Wang M. Yang S. Zheng Y. (2021). Global, regional, and national burden of diabetes-related chronic kidney disease from 1990 to 2019. Front. Endocrinol. (Lausanne) 12, 672350. 10.3389/fendo.2021.672350 34276558 PMC8281340

[B21] Dua T. K. Joardar S. Chakraborty P. Bhowmick S. Saha A. De Feo V. (2021). Myricitrin, a glycosyloxyflavone in myrica esculenta bark ameliorates diabetic nephropathy via improving glycemic status, reducing oxidative stress, and suppressing inflammation. Molecules 26, 258. 10.3390/molecules26020258 33419120 PMC7825565

[B22] Eckerdt F. Yuan J. Saxena K. Martin B. Kappel S. Lindenau C. (2005). Polo-like kinase 1-mediated phosphorylation stabilizes Pin1 by inhibiting its ubiquitination in human cells. J. Biol. Chem. 280, 36575–36583. 10.1074/jbc.M504548200 16118204

[B23] Efrati S. Berman S. Tov Y. S. Averbukh Z. Weissgarten J. (2009). Hyperglycemia alters renal cell responsiveness to pressure in a model of malignant hypertension. J. Hypertens. 27, 365–375. 10.1097/HJH.0b013e32831b46ab 19155791

[B24] Esnault S. Braun R. K. Shen Z. J. Xiang Z. Heninger E. Love R. B. (2007). Pin1 modulates the type 1 immune response. PLoS One 2, e226. 10.1371/journal.pone.0000226 17311089 PMC1790862

[B25] Feng D. Yao J. Wang G. Li Z. Zu G. Li Y. (2017). Inhibition of p66Shc-mediated mitochondrial apoptosis via targeting prolyl-isomerase Pin1 attenuates intestinal ischemia/reperfusion injury in rats. Clin. Sci. (Lond) 131, 759–773. 10.1042/CS20160799 28232511

[B26] Fischer G. Tradler T. Zarnt T. (1998). The mode of action of peptidyl prolyl cis/trans isomerases *in vivo*: binding vs catalysis. FEBS Lett. 426, 17–20. 10.1016/s0014-5793(98)00242-7 9598969

[B27] Fischer G. Wittmann-Liebold B. Lang K. Kiefhaber T. Schmid F. X. (1989). Cyclophilin and peptidyl-prolyl cis-trans isomerase are probably identical proteins. Nature 337, 476–478. 10.1038/337476a0 2492638

[B28] Fraser W. D. (2009). Hyperparathyroidism. Lancet 374, 145–158. 10.1016/S0140-6736(09)60507-9 19595349

[B29] Galas M. C. Dourlen P. Begard S. Ando K. Blum D. Hamdane M. (2006). The peptidylprolyl cis/trans-isomerase Pin1 modulates stress-induced dephosphorylation of Tau in neurons. Implication in a pathological mechanism related to Alzheimer disease. J. Biol. Chem. 281, 19296–19304. 10.1074/jbc.M601849200 16675464

[B30] Galat A. (2003). Peptidylprolyl cis/trans isomerases (immunophilins): biological diversity--targets--functions. Curr. Top. Med. Chem. 3, 1315–1347. 10.2174/1568026033451862 12871165

[B31] Guo C. Hou X. Dong L. Marakovits J. Greasley S. Dagostino E. (2014). Structure-based design of novel human Pin1 inhibitors (III): optimizing affinity beyond the phosphate recognition pocket. Bioorg Med. Chem. Lett. 24, 4187–4191. 10.1016/j.bmcl.2014.07.044 25091930

[B32] Guo F. Zhuang X. Han M. Lin W. (2020). Polysaccharides from Enteromorpha prolifera protect against carbon tetrachloride-induced acute liver injury in mice via activation of Nrf2/HO-1 signaling, and suppression of oxidative stress, inflammation and apoptosis. Food Funct. 11, 4485–4498. 10.1039/d0fo00575d 32378684

[B33] Hanes S. D. Shank P. R. Bostian K. A. (1989). Sequence and mutational analysis of ESS1, a gene essential for growth in *Saccharomyces cerevisiae* . Yeast 5, 55–72. 10.1002/yea.320050108 2648698

[B34] Hani J. Stumpf G. Domdey H. (1995). PTF1 encodes an essential protein in *Saccharomyces cerevisiae*, which shows strong homology with a new putative family of PPIases. FEBS Lett. 365, 198–202. 10.1016/0014-5793(95)00471-k 7781779

[B35] Hassan A. Khalaily N. Kilav-Levin R. Nechama M. Volovelsky O. Silver J. (2022b). Molecular mechanisms of parathyroid disorders in chronic kidney disease. Metabolites 12, 111. 10.3390/metabo12020111 35208186 PMC8878033

[B36] Hassan A. Pollak Y. E. Kilav-Levin R. Silver J. London N. Nechama M. (2022a). Kidney failure alters parathyroid Pin1 phosphorylation and parathyroid hormone mRNA-binding proteins, leading to secondary hyperparathyroidism. J. Am. Soc. Nephrol. 33, 1677–1693. 10.1681/ASN.2022020197 35961788 PMC9529182

[B37] He J. Xu Y. Koya D. Kanasaki K. (2013). Role of the endothelial-to-mesenchymal transition in renal fibrosis of chronic kidney disease. Clin. Exp. Nephrol. 17, 488–497. 10.1007/s10157-013-0781-0 23430391

[B38] Hennig L. Christner C. Kipping M. Schelbert B. Rucknagel K. P. Grabley S. (1998). Selective inactivation of parvulin-like peptidyl-prolyl cis/trans isomerases by juglone. Biochemistry 37, 5953–5960. 10.1021/bi973162p 9558330

[B39] Himmelfarb J. Vanholder R. Mehrotra R. Tonelli M. (2020). The current and future landscape of dialysis. Nat. Rev. Nephrol. 16, 573–585. 10.1038/s41581-020-0315-4 32733095 PMC7391926

[B40] Huang L. Mo Z. Li S. Qin X. (2018). The association between PIN1 genetic polymorphisms and the risk of chronic hepatitis B and hepatitis B virus-related liver cirrhosis: a case-control study. Med. Baltim. 97, e12123. 10.1097/MD.0000000000012123 PMC6392626 30170446

[B41] Inoue M. K. Matsunaga Y. Nakatsu Y. Yamamotoya T. Ueda K. Kushiyama A. (2019). Possible involvement of normalized Pin1 expression level and AMPK activation in the molecular mechanisms underlying renal protective effects of SGLT2 inhibitors in mice. Diabetol. Metab. Syndr. 11, 57. 10.1186/s13098-019-0454-6 31367234 PMC6647324

[B42] Jordens J. Janssens V. Longin S. Stevens I. Martens E. Bultynck G. (2006). The protein phosphatase 2A phosphatase activator is a novel peptidyl-prolyl cis/trans-isomerase. J. Biol. Chem. 281, 6349–6357. 10.1074/jbc.M507760200 16380387

[B43] Joshi G. Aluise C. D. Cole M. P. Sultana R. Pierce W. M. Vore M. (2010). Alterations in brain antioxidant enzymes and redox proteomic identification of oxidized brain proteins induced by the anti-cancer drug adriamycin: implications for oxidative stress-mediated chemobrain. Neuroscience 166, 796–807. 10.1016/j.neuroscience.2010.01.021 20096337 PMC2852883

[B44] Kato H. Naito M. Saito T. Hideyama T. Suzuki Y. Kimura T. (2022). Prolyl isomerase Pin1 expression in the spinal motor neurons of patients with sporadic amyotrophic lateral sclerosis. J. Clin. Neurol. 18, 463–469. 10.3988/jcn.2022.18.4.463 35796272 PMC9262457

[B45] Kilav-Levin R. Hassan A. Nechama M. Shilo V. Silver J. Ben-Dov I. Z. (2020). Post-transcriptional mechanisms regulating parathyroid hormone gene expression in secondary hyperparathyroidism. FEBS J. 287, 2903–2913. 10.1111/febs.15300 32191397

[B46] Kim G. Khanal P. Kim J. Y. Yun H. J. Lim S. C. Shim J. H. (2015). COT phosphorylates prolyl-isomerase Pin1 to promote tumorigenesis in breast cancer. Mol. Carcinog. 54, 440–448. 10.1002/mc.22112 24265246

[B47] Kim H. J. Cho Y. A. Lee Y. M. Lee S. Y. Bae W. J. Kim E. C. (2016). PIN1 suppresses the hepatic differentiation of pulp stem cells via Wnt3a. J. Dent. Res. 95, 1415–1424. 10.1177/0022034516659642 27439725

[B48] Kozono S. Lin Y. M. Seo H. S. Pinch B. Lian X. Qiu C. (2018). Arsenic targets Pin1 and cooperates with retinoic acid to inhibit cancer-driving pathways and tumor-initiating cells. Nat. Commun. 9, 3069. 10.1038/s41467-018-05402-2 30093655 PMC6085299

[B49] Kumar R. (2009). Pin1 regulates parathyroid hormone mRNA stability. J. Clin. Invest. 119, 2887–2891. 10.1172/JCI40784 19770518 PMC2752089

[B50] Lang K. Schmid F. X. Fischer G. (1987). Catalysis of protein folding by prolyl isomerase. Nature 329, 268–270. 10.1038/329268a0 3306408

[B51] Lederer E. Ouseph R. (2007). Chronic kidney disease. Am. J. Kidney Dis. 49, 162–171. 10.1053/j.ajkd.2006.09.021 17185158

[B52] Lee T. H. Chen C. H. Suizu F. Huang P. Schiene-Fischer C. Daum S. (2011). Death-associated protein kinase 1 phosphorylates Pin1 and inhibits its prolyl isomerase activity and cellular function. Mol. Cell 42, 147–159. 10.1016/j.molcel.2011.03.005 21497122 PMC3088080

[B53] Levey A. S. Coresh J. Balk E. Kausz A. T. Levin A. Steffes M. W. (2003). National Kidney Foundation practice guidelines for chronic kidney disease: evaluation, classification, and stratification. Ann. Intern Med. 139, 137–147. 10.7326/0003-4819-139-2-200307150-00013 12859163

[B54] Levey A. S. James M. T. (2017). Acute kidney injury. Ann. Intern Med. 167, ITC66-ITC80–ITC80. 10.7326/AITC201711070 29114754

[B55] Li X. Hyink D. P. Radbill B. Sudol M. Zhang H. Zheleznova N. N. (2009). Protein kinase-X interacts with Pin-1 and Polycystin-1 during mouse kidney development. Kidney Int. 76, 54–62. 10.1038/ki.2009.95 19367327

[B56] Liang E. S. Cheng W. Yang R. X. Bai W. W. Liu X. Zhao Y. X. (2018). Peptidyl-prolyl isomerase Pin1 deficiency attenuates angiotensin II-induced abdominal aortic aneurysm formation in ApoE(-/-) mice. J. Mol. Cell Cardiol. 114, 334–344. 10.1016/j.yjmcc.2017.12.006 29269260

[B57] Lim H. J. Shim S. B. Jee S. W. Lee S. H. Lim C. J. Hong J. T. (2013). Green tea catechin leads to global improvement among Alzheimer's disease-related phenotypes in NSE/hAPP-C105 Tg mice. J. Nutr. Biochem. 24, 1302–1313. 10.1016/j.jnutbio.2012.10.005 23333093

[B58] Lim Y. B. Park T. J. Lim I. K. (2008). B cell translocation gene 2 enhances susceptibility of HeLa cells to doxorubicin-induced oxidative damage. J. Biol. Chem. 283, 33110–33118. 10.1074/jbc.M804255200 18840609 PMC2662249

[B59] Lin Y. C. Lu L. T. Chen H. Y. Duan X. Lin X. Feng X. H. (2014). SCP phosphatases suppress renal cell carcinoma by stabilizing PML and inhibiting mTOR/HIF signaling. Cancer Res. 74, 6935–6946. 10.1158/0008-5472.CAN-14-1330 25293974

[B60] Linehan W. M. Ricketts C. J. (2019). The Cancer Genome Atlas of renal cell carcinoma: findings and clinical implications. Nat. Rev. Urol. 16, 539–552. 10.1038/s41585-019-0211-5 31278395

[B61] Liou Y. C. Sun A. Ryo A. Zhou X. Z. Yu Z. X. Huang H. K. (2003). Role of the prolyl isomerase Pin1 in protecting against age-dependent neurodegeneration. Nature 424, 556–561. 10.1038/nature01832 12891359

[B62] Liu X. Liang E. Song X. Du Z. Zhang Y. Zhao Y. (2016). Inhibition of Pin1 alleviates myocardial fibrosis and dysfunction in STZ-induced diabetic mice. Biochem. Biophys. Res. Commun. 479, 109–115. 10.1016/j.bbrc.2016.09.050 27634219

[B63] Liu Y. (2011). Cellular and molecular mechanisms of renal fibrosis. Nat. Rev. Nephrol. 7, 684–696. 10.1038/nrneph.2011.149 22009250 PMC4520424

[B64] Liu Y. Zhao D. Qiu F. Zhang L. L. Liu S. K. Li Y. Y. (2017). Manipulating PML SUMOylation via silencing UBC9 and RNF4 regulates cardiac fibrosis. Mol. Ther. 25, 666–678. 10.1016/j.ymthe.2016.12.021 28143738 PMC5363217

[B65] Lu J. Hu Z. Wei S. Wang L. E. Liu Z. El-Naggar A. K. (2009). A novel functional variant (-842G>C) in the PIN1 promoter contributes to decreased risk of squamous cell carcinoma of the head and neck by diminishing the promoter activity. Carcinogenesis 30, 1717–1721. 10.1093/carcin/bgp171 19625347 PMC2757547

[B66] Lu K. P. Hanes S. D. Hunter T. (1996). A human peptidyl-prolyl isomerase essential for regulation of mitosis. Nature 380, 544–547. 10.1038/380544a0 8606777

[B67] Lu K. P. Zhou X. Z. (2007). The prolyl isomerase PIN1: a pivotal new twist in phosphorylation signalling and disease. Nat. Rev. Mol. Cell Biol. 8, 904–916. 10.1038/nrm2261 17878917

[B68] Lu P. J. Zhou X. Z. Liou Y. C. Noel J. P. Lu K. P. (2002). Critical role of WW domain phosphorylation in regulating phosphoserine binding activity and Pin1 function. J. Biol. Chem. 277, 2381–2384. 10.1074/jbc.C100228200 11723108

[B69] Luyckx V. A. Al-Aly Z. Bello A. K. Bellorin-Font E. Carlini R. G. Fabian J. (2021). Sustainable Development Goals relevant to kidney health: an update on progress. Nat. Rev. Nephrol. 17, 15–32. 10.1038/s41581-020-00363-6 33188362 PMC7662029

[B70] Lv L. Ye M. Duan R. Yuan K. Chen J. Liang W. (2018). Downregulation of Pin1 in human atherosclerosis and its association with vascular smooth muscle cell senescence. J. Vasc. Surg. 68, 873–883. 10.1016/j.jvs.2017.09.006 28986099

[B71] Lv Z. Zhang Y. Cao H. Liu Q. Feng X. Yin H. (2022). PIN1 protects auditory hair cells from senescence via autophagy. PeerJ 10, e14267. 10.7717/peerj.14267 36340199 PMC9635358

[B72] Matena A. Rehic E. Honig D. Kamba B. Bayer P. (2018). Structure and function of the human parvulins Pin1 and Par14/17. Biol. Chem. 399, 101–125. 10.1515/hsz-2017-0137 29040060

[B73] Nakatsu Y. Matsunaga Y. Yamamotoya T. Ueda K. Inoue Y. Mori K. (2016). Physiological and pathogenic roles of prolyl isomerase Pin1 in metabolic regulations via multiple signal transduction pathway modulations. Int. J. Mol. Sci. 17, 1495. 10.3390/ijms17091495 27618008 PMC5037772

[B74] Nakatsu Y. Otani Y. Sakoda H. Zhang J. Guo Y. Okubo H. (2012). Role of Pin1 protein in the pathogenesis of nonalcoholic steatohepatitis in a rodent model. J. Biol. Chem. 287, 44526–44535. 10.1074/jbc.M112.397133 23112047 PMC3531766

[B75] Namgoong G. M. Khanal P. Cho H. G. Lim S. C. Oh Y. K. Kang B. S. (2010). The prolyl isomerase Pin1 induces LC-3 expression and mediates tamoxifen resistance in breast cancer. J. Biol. Chem. 285, 23829–23841. 10.1074/jbc.M109.092874 20479004 PMC2911270

[B76] Nechama M. Uchida T. Mor Yosef-Levi I. Silver J. Naveh-Many T. (2009). The peptidyl-prolyl isomerase Pin1 determines parathyroid hormone mRNA levels and stability in rat models of secondary hyperparathyroidism. J. Clin. Invest. 119, 3102–3114. 10.1172/JCI39522 19770516 PMC2752082

[B77] Nigro P. Pompilio G. Capogrossi M. C. (2013). Cyclophilin A: a key player for human disease. Cell Death Dis. 4, e888. 10.1038/cddis.2013.410 24176846 PMC3920964

[B78] Nozaki Y. Fujita K. Yoneda M. Wada K. Shinohara Y. Takahashi H. (2009). Long-term combination therapy of ezetimibe and acarbose for non-alcoholic fatty liver disease. J. Hepatol. 51, 548–556. 10.1016/j.jhep.2009.05.017 19596472

[B79] Oke S. L. Sohi G. Hardy D. B. (2020). Perinatal protein restriction with postnatal catch-up growth leads to elevated p66Shc and mitochondrial dysfunction in the adult rat liver. Reproduction 159, 27–39. 10.1530/REP-19-0188 31689235 PMC6933810

[B80] O Toole J. F. Sedor J. R. (2014). Kidney disease: new technologies translate mechanisms to cure. J. Clin. Invest. 124, 2294–2298. 10.1172/JCI76825 24892702 PMC4089455

[B81] Paneni F. Costantino S. Castello L. Battista R. Capretti G. Chiandotto S. (2015). Targeting prolyl-isomerase Pin1 prevents mitochondrial oxidative stress and vascular dysfunction: insights in patients with diabetes. Eur. Heart J. 36, 817–828. 10.1093/eurheartj/ehu179 24801072

[B82] Park J. E. Lee J. A. Park S. G. Lee D. H. Kim S. J. Kim H. J. (2012). A critical step for JNK activation: isomerization by the prolyl isomerase Pin1. Cell Death Differ. 19, 153–161. 10.1038/cdd.2011.82 21660049 PMC3252824

[B83] Patel D. D. Parchwani D. Vachhani U. Parchwani T. Raghavani P. Rajput A. (2022). A molecular insight of the role of PIN-1 promoter polymorphism (- 667C > T; rs2233679) in chronic kidney disease patients with secondary hyperparathyroidism. Indian J. Clin. Biochem. 37, 319–327. 10.1007/s12291-021-00997-8 35873609 PMC9300778

[B84] Peng Y. Zhou G. Guo M. Cheng Z. Luo S. Guo Y. (2023). Inhibition of stimulator of interferon genes protects against myocardial ischemia-reperfusion injury in diabetic mice. Cardiovasc Innov. Appl. 8. 10.15212/cvia.2023.0020

[B85] Pinton P. Rimessi A. Marchi S. Orsini F. Migliaccio E. Giorgio M. (2007). Protein kinase C beta and prolyl isomerase 1 regulate mitochondrial effects of the life-span determinant p66Shc. Science 315, 659–663. 10.1126/science.1135380 17272725

[B86] Pu W. Li J. Zheng Y. Shen X. Fan X. Zhou J. K. (2018). Targeting Pin1 by inhibitor API-1 regulates microRNA biogenesis and suppresses hepatocellular carcinoma development. Hepatology 68, 547–560. 10.1002/hep.29819 29381806

[B87] Qiu F. Dong C. Liu Y. Shao X. Huang D. Han Y. (2018). Pharmacological inhibition of SUMO-1 with ginkgolic acid alleviates cardiac fibrosis induced by myocardial infarction in mice. Toxicol. Appl. Pharmacol. 345, 1–9. 10.1016/j.taap.2018.03.006 29524504

[B88] Rangasamy V. Mishra R. Sondarva G. Das S. Lee T. H. Bakowska J. C. (2012). Mixed-lineage kinase 3 phosphorylates prolyl-isomerase Pin1 to regulate its nuclear translocation and cellular function. Proc. Natl. Acad. Sci. U. S. A. 109, 8149–8154. 10.1073/pnas.1200804109 22566623 PMC3361382

[B89] Reese S. Vidyasagar A. Jacobson L. Acun Z. Esnault S. Hullett D. (2010). The Pin 1 inhibitor juglone attenuates kidney fibrogenesis via Pin 1-independent mechanisms in the unilateral ureteral occlusion model. Fibrogenes. Tissue Repair 3, 1. 10.1186/1755-1536-3-1 PMC2823698 20047646

[B90] Robinson R. A. Lange M. B. Sultana R. Galvan V. Fombonne J. Gorostiza O. (2011). Differential expression and redox proteomics analyses of an Alzheimer disease transgenic mouse model: effects of the amyloid-β peptide of amyloid precursor protein. Neuroscience 177, 207–222. 10.1016/j.neuroscience.2011.01.005 21223993 PMC3058851

[B91] Rostam M. A. Piva T. J. Rezaei H. B. Kamato D. Little P. J. Zheng W. (2015). Peptidyl-prolyl isomerases: functionality and potential therapeutic targets in cardiovascular disease. Clin. Exp. Pharmacol. Physiol. 42, 117–124. 10.1111/1440-1681.12335 25377120

[B92] Rudrabhatla P. Zheng Y. L. Amin N. D. Kesavapany S. Albers W. Pant H. C. (2008). Pin1-dependent prolyl isomerization modulates the stress-induced phosphorylation of high molecular weight neurofilament protein. J. Biol. Chem. 283, 26737–26747. 10.1074/jbc.M801633200 18635547 PMC2546547

[B93] Ryo A. Liou Y. C. Wulf G. Nakamura M. Lee S. W. Lu K. P. (2002). PIN1 is an E2F target gene essential for Neu/Ras-induced transformation of mammary epithelial cells. Mol. Cell Biol. 22, 5281–5295. 10.1128/mcb.22.15.5281-5295.2002 12101225 PMC133940

[B94] Ryo A. Nakamura M. Wulf G. Liou Y. C. Lu K. P. (2001). Pin1 regulates turnover and subcellular localization of beta-catenin by inhibiting its interaction with APC. Nat. Cell Biol. 3, 793–801. 10.1038/ncb0901-793 11533658

[B95] Saeed U. Piracha Z. Z. (2023). PIN1 and PIN4 inhibition via parvulin impeders Juglone, PiB, ATRA, 6,7,4'-THIF, KPT6566, and EGCG thwarted hepatitis B virus replication. Front. Microbiol. 14, 921653. 10.3389/fmicb.2023.921653 36760500 PMC9905731

[B96] Sakai S. Shimojo N. Kimura T. Tajiri K. Maruyama H. Homma S. (2014). Involvement of peptidyl-prolyl isomerase Pin1 in the inhibitory effect of fluvastatin on endothelin-1-induced cardiomyocyte hypertrophy. Life Sci. 102, 98–104. 10.1016/j.lfs.2014.03.018 24657892

[B97] Schmid F. X. (1993). Prolyl isomerase: enzymatic catalysis of slow protein-folding reactions. Annu. Rev. Biophys. Biomol. Struct. 22, 123–142. 10.1146/annurev.bb.22.060193.001011 7688608

[B98] Seckler R. Jaenicke R. (1992). Protein folding and protein refolding. FASEB J. 6, 2545–2552. 10.1096/fasebj.6.8.1592207 1592207

[B99] Shen Z. J. Braun R. K. Hu J. Xie Q. Chu H. Love R. B. (2012). Pin1 protein regulates Smad protein signaling and pulmonary fibrosis. J. Biol. Chem. 287, 23294–23305. 10.1074/jbc.M111.313684 22613712 PMC3390608

[B100] Shen Z. J. Esnault S. Rosenthal L. A. Szakaly R. J. Sorkness R. L. Westmark P. R. (2008). Pin1 regulates TGF-beta1 production by activated human and murine eosinophils and contributes to allergic lung fibrosis. J. Clin. Invest. 118, 479–490. 10.1172/JCI32789 18188456 PMC2176187

[B101] Shen Z. J. Hu J. Shiizaki K. Kuro-o M. Malter J. S. (2016). Phosphate-induced renal fibrosis requires the prolyl isomerase Pin1. PLoS One 11, e0150093. 10.1371/journal.pone.0150093 26914452 PMC4767802

[B102] Smet C. Wieruszeski J. M. Buee L. Landrieu I. Lippens G. (2005). Regulation of Pin1 peptidyl-prolyl cis/trans isomerase activity by its WW binding module on a multi-phosphorylated peptide of Tau protein. FEBS Lett. 579, 4159–4164. 10.1016/j.febslet.2005.06.048 16024016

[B103] So K. Y. Ahn S. G. Oh S. H. (2015). Autophagy regulated by prolyl isomerase Pin1 and phospho-Ser-GSK3αβ involved in protection of oral squamous cell carcinoma against cadmium toxicity. Biochem. Biophys. Res. Commun. 466, 541–546. 10.1016/j.bbrc.2015.09.066 26381174

[B104] So K. Y. Oh S. H. (2015). Prolyl isomerase Pin1 regulates cadmium-induced autophagy via ubiquitin-mediated post-translational stabilization of phospho-Ser GSK3αβ in human hepatocellular carcinoma cells. Biochem. Pharmacol. 98, 511–521. 10.1016/j.bcp.2015.09.007 26445356

[B105] Struckmann K. Schraml P. Simon R. Elmenhorst K. Mirlacher M. Kononen J. (2004). Impaired expression of the cell cycle regulator BTG2 is common in clear cell renal cell carcinoma. Cancer Res. 64, 1632–1638. 10.1158/0008-5472.can-03-1687 14996721

[B106] Sultana R. Boyd-Kimball D. Poon H. F. Cai J. Pierce W. M. Klein J. B. (2006). Oxidative modification and down-regulation of Pin1 in Alzheimer's disease hippocampus: a redox proteomics analysis. Neurobiol. Aging 27, 918–925. 10.1016/j.neurobiolaging.2005.05.005 15950321

[B107] Sun L. Xiao L. Nie J. Liu F. Y. Ling G. H. Zhu X. J. (2010). p66Shc mediates high-glucose and angiotensin II-induced oxidative stress renal tubular injury via mitochondrial-dependent apoptotic pathway. Am. J. Physiol. Ren. Physiol. 299, F1014–F1025. 10.1152/ajprenal.00414.2010 PMC2980400 20739391

[B108] Sun Q. Fan G. Zhuo Q. Dai W. Ye Z. Ji S. (2020). Pin1 promotes pancreatic cancer progression and metastasis by activation of NF-κB-IL-18 feedback loop. Cell Prolif. 53, e12816. 10.1111/cpr.12816 32347623 PMC7260075

[B109] Sun X. Li Z. Wang L. Wang Y. Lu C. (2023). Berberine ameliorates diabetic cardiomyopathy in mice by decreasing cardiomyocyte apoptosis and oxidative stress. Cardiovasc Innov. Appl. 8. 10.15212/cvia.2023.0064

[B110] Teng B. L. Hacker K. E. Chen S. Means A. R. Rathmell W. K. (2011). Tumor suppressive activity of prolyl isomerase Pin1 in renal cell carcinoma. Mol. Oncol. 5, 465–474. 10.1016/j.molonc.2011.06.002 21764651 PMC3194764

[B111] Thorpe J. R. Morley S. J. Rulten S. L. (2001). Utilizing the peptidyl-prolyl cis-trans isomerase pin1 as a probe of its phosphorylated target proteins. Examples of binding to nuclear proteins in a human kidney cell line and to tau in Alzheimer's diseased brain. J. Histochem Cytochem 49, 97–108. 10.1177/002215540104900110 11118482

[B112] Tycova I. Sulkova S. D. Stepankova J. Krejcik Z. Merkerova M. D. Stranecky V. (2016). Molecular patterns of diffuse and nodular parathyroid hyperplasia in long-term hemodialysis. Am. J. Physiol. Endocrinol. Metab. 311, E720-E729–E729. 10.1152/ajpendo.00517.2015 27600827

[B113] Urusova D. V. Shim J. H. Kim D. J. Jung S. K. Zykova T. A. Carper A. (2011). Epigallocatechin-gallate suppresses tumorigenesis by directly targeting Pin1. Cancer Prev. Res. (Phila) 4, 1366–1377. 10.1158/1940-6207.CAPR-11-0301 21750208 PMC3244823

[B114] Verdecia M. A. Bowman M. E. Lu K. P. Hunter T. Noel J. P. (2000). Structural basis for phosphoserine-proline recognition by group IV WW domains. Nat. Struct. Biol. 7, 639–643. 10.1038/77929 10932246

[B115] Wang Y. Li Z. Zhang Y. Yang W. Sun J. Shan L. (2016). Targeting Pin1 protects mouse cardiomyocytes from high-dose alcohol-induced apoptosis. Oxid. Med. Cell Longev. 2016, 4528906. 10.1155/2016/4528906 26697133 PMC4678095

[B116] Warnke S. Baldauf C. Bowers M. T. Pagel K. von Helden G. (2014). Photodissociation of conformer-selected ubiquitin ions reveals site-specific cis/trans isomerization of proline peptide bonds. J. Am. Chem. Soc. 136, 10308–10314. 10.1021/ja502994b 25007274

[B117] Wei S. Kozono S. Kats L. Nechama M. Li W. Guarnerio J. (2015). Active Pin1 is a key target of all-trans retinoic acid in acute promyelocytic leukemia and breast cancer. Nat. Med. 21, 457–466. 10.1038/nm.3839 25849135 PMC4425616

[B118] Wettersten H. I. Aboud O. A. Lara P. N. Jr. Weiss R. H. (2017). Metabolic reprogramming in clear cell renal cell carcinoma. Nat. Rev. Nephrol. 13, 410–419. 10.1038/nrneph.2017.59 28480903

[B119] Wintjens R. Wieruszeski J. M. Drobecq H. Rousselot-Pailley P. Buee L. Lippens G. (2001). 1H NMR study on the binding of Pin1 Trp-Trp domain with phosphothreonine peptides. J. Biol. Chem. 276, 25150–25156. 10.1074/jbc.M010327200 11313338

[B120] Wu D. Huang D. Li L. L. Ni P. Li X. X. Wang B. (2019). TGF-β1-PML SUMOylation-peptidyl-prolyl cis-trans isomerase NIMA-interacting 1 (Pin1) form a positive feedback loop to regulate cardiac fibrosis. J. Cell Physiol. 234, 6263–6273. 10.1002/jcp.27357 30246389

[B121] Wu X. Li M. Chen S. Q. Li S. Guo F. (2018). Pin1 facilitates isoproterenol-induced cardiac fibrosis and collagen deposition by promoting oxidative stress and activating the MEK1/2-ERK1/2 signal transduction pathway in rats. Int. J. Mol. Med. 41, 1573–1583. 10.3892/ijmm.2017.3354 29286102 PMC5819929

[B122] Xu Y. X. Manley J. L. (2007). The prolyl isomerase Pin1 functions in mitotic chromosome condensation. Mol. Cell 26, 287–300. 10.1016/j.molcel.2007.03.020 17466629

[B123] Yaffe M. B. Schutkowski M. Shen M. Zhou X. Z. Stukenberg P. T. Rahfeld J. U. (1997). Sequence-specific and phosphorylation-dependent proline isomerization: a potential mitotic regulatory mechanism. Science 278, 1957–1960. 10.1126/science.278.5345.1957 9395400

[B124] Yan M. T. Chao C. T. Lin S. H. (2021). Chronic kidney disease: strategies to retard progression. Int. J. Mol. Sci. 22, 10084. 10.3390/ijms221810084 34576247 PMC8470895

[B125] Yang J. W. Hien T. T. Lim S. C. Jun D. W. Choi H. S. Yoon J. H. (2014). Pin1 induction in the fibrotic liver and its roles in TGF-β1 expression and Smad2/3 phosphorylation. J. Hepatol. 60, 1235–1241. 10.1016/j.jhep.2014.02.004 24530597

[B126] Yang L. Li J. Zang G. Song S. Sun Z. Li X. (2023). Pin1/YAP pathway mediates matrix stiffness-induced epithelial-mesenchymal transition driving cervical cancer metastasis via a non-Hippo mechanism. Bioeng. Transl. Med. 8, e10375. 10.1002/btm2.10375 36684109 PMC9842039

[B127] Yeh E. S. Lew B. O. Means A. R. (2006). The loss of PIN1 deregulates cyclin E and sensitizes mouse embryo fibroblasts to genomic instability. J. Biol. Chem. 281, 241–251. 10.1074/jbc.M505770200 16223725

[B128] Yeh E. S. Means A. R. (2007). PIN1, the cell cycle and cancer. Nat. Rev. Cancer 7, 381–388. 10.1038/nrc2107 17410202

[B129] Yu H. Jiang G. Hu W. Xu C. (2022). Pin1 aggravates renal injury induced by ischemia and reperfusion in rats via Nrf2/HO-1 mediated endoplasmic reticulum stress. Acta Cir. Bras. 37, e370101. 10.1590/acb370101 35416857 PMC9000979

[B130] Zhang H. He Z. Deng P. Lu M. Zhou C. Yang L. (2022b). PIN1-mediated ROS production is involved in antagonism of N-acetyl-L-cysteine against arsenic-induced hepatotoxicity. Toxicol. Res. (Camb) 11, 628–643. 10.1093/toxres/tfac040 36051664 PMC9424717

[B131] Zhang J. H. Ni S. Y. Tan Y. T. Luo J. Wang S. C. (2022a). A bibliometric analysis of PIN1 and cell death. Front. Cell Dev. Biol. 10, 1043725. 10.3389/fcell.2022.1043725 36393861 PMC9659740

[B132] Zhang M. Lin L. Xu C. Chai D. Peng F. Lin J. (2018). VDR agonist prevents diabetic endothelial dysfunction through inhibition of prolyl isomerase-1-mediated mitochondrial oxidative stress and inflammation. Oxid. Med. Cell Longev. 2018, 1714896. 10.1155/2018/1714896 29849865 PMC5925189

[B133] Zhang X. L. Zhang G. Bai Z. H. (2021). miR-34a attenuates myocardial fibrosis in diabetic cardiomyopathy mice via targeting Pin-1. Cell Biol. Int. 45, 642–653. 10.1002/cbin.11512 33289184

[B134] Zhao H. Cui G. Jin J. Chen X. Xu B. (2016). Synthesis and Pin1 inhibitory activity of thiazole derivatives. Bioorg Med. Chem. 24, 5911–5920. 10.1016/j.bmc.2016.09.049 27692510

[B135] Zhao X. Wang D. Wan S. Liu X. Wang W. Wang L. (2021). The suppression of pin1-alleviated oxidative stress through the p38 MAPK pathway in ischemia- and reperfusion-induced acute kidney injury. Oxid. Med. Cell Longev. 2021, 1313847. 10.1155/2021/1313847 34373763 PMC8349297

[B136] Zhao Y. Zhang L. L. Ding F. X. Cao P. Qi Y. Y. Wang J. (2017). Pin1 and secondary hyperparathyroidism of chronic kidney disease: gene polymorphisms and protein levels. Ren. Fail 39, 159–165. 10.1080/0886022X.2016.1256310 27876426 PMC6014329

[B137] Zhou X. Z. Lu K. P. (2016). The isomerase PIN1 controls numerous cancer-driving pathways and is a unique drug target. Nat. Rev. Cancer 16, 463–478. 10.1038/nrc.2016.49 27256007

[B138] Zhu M. Chen J. Wen M. Sun Z. Sun X. Wang J. (2014). Propofol protects against angiotensin II-induced mouse hippocampal HT22 cells apoptosis via inhibition of p66Shc mitochondrial translocation. Neuromolecular Med. 16, 772–781. 10.1007/s12017-014-8326-6 25151272

[B139] Zhu X. Xu X. Du C. Su Y. Yin L. Tan X. (2022). An examination of the protective effects and molecular mechanisms of curcumin, a polyphenol curcuminoid in diabetic nephropathy. Biomed. Pharmacother. 153, 113438. 10.1016/j.biopha.2022.113438 36076553

